# Mystixin-7 Peptide Protects Ionotropic Glutamatergic Mechanisms against Glutamate-Induced Excitotoxicity* In Vitro*

**DOI:** 10.1155/2016/5151843

**Published:** 2016-07-18

**Authors:** Anatoly A. Mokrushin

**Affiliations:** Laboratory for Regulation of Brain Neurons Functions, I. P. Pavlov Institute of Physiology, Russian Academy of Sciences, Nab. Makarova 6, Saint Petersburg 199034, Russia

## Abstract

Hyperactivation of the* N*-methyl-D-aspartic acid type glutamate receptors (NMDARs) causes glutamate excitotoxicity, a process potentially important for many neurological diseases. This study aims to investigate protective effects of the synthetic corticotrophin-releasing factor-like peptide, mystixin-7 (MTX), on model glutamate-induced excitotoxicity* in vitro*. The technique online monitoring of electrophysiological parameters (excitatory glutamatergic alpha-amino-3-hydroxy-5-methyl-4-isoxazolepropionic (AMPAR) and NMDAR-dependent postsynaptic mechanisms) in the olfactory cortex slices was used. Application of* L*-glutamate in toxic concentration (20 mM) on slices evoked hyperactivation of NMDARs and weaker activation of the AMPARs. Upon further action agonist, the excessive activation of glutamate receptors was replaced by their irreversible blockade. Pretreatment of the slices using MTX in different concentrations (50 and 100 mg/mL) protected both NMDARs and AMPARs from glutamate-induced damage. An enzymatic treatment of MTX reduced hyperactivation of both NMDARs and AMPARs. The present study demonstrated that MTX minipeptide protected the functioning of both NMDARs and AMPARs against glutamate-induced damage. The MTX peptide is a prospective candidate for elaborated medication in treatment of neurological diseases.

## 1. Introduction

Glutamate is a major excitatory neurotransmitter in the central nervous system of mammals. This substance plays a key role in various adaptive functions, including learning, memory, emotional reactivity, sensory perception, and control of locomotion [1–3]. However, powerful stress (e.g., trauma, ischemia, epilepsy, and anoxia) induces massive release of intracellular glutamate into the extracellular space [4]. It results in hyperactivation of glutamate receptors, impairment of glutamate reuptake, and an excessive influx of Ca^2+^ entry into cells [5]. Increased intracellular Ca^2+^ levels activate a multitude of potentially neurotoxic mechanisms, such as the early induction of a calcium-dependent protease, calpain, which cleaves intracellular structural proteins such as spectrin, causing the collapse of intracellular structures and cell death [6].

Glutamate excitotoxicity actively not only is involved in acute injuries, as hypoxia, ischemia, hypoglycemia, and epileptic seizures, but also has been related to a wide range of neurological disorders, as Alzheimer's, Huntington's, and Parkinson's diseases, amyotrophic lateral sclerosis, and schizophrenia and other psychiatric disorders [3, 7–9]. Consequently, an important and practically significant problem of modern neurobiology is in searching reliable, effective medicines without side effects, which protect the functioning of glutamatergic mechanisms.

However, since glutamate is the major excitatory transmitter in the whole brain, generalized inhibition of a glutamate receptors subtype like the* N*-methyl-D-aspartic acid receptors (NMDARs) induces side effects that clearly limit the potential for clinical applications [10–13]. It is believed that chemical molecules with a relatively fast diffusion rate, and which only transiently and reversibly block NMDARs, promise to be potential protective drug candidates against excitotoxicity-evoked brain damage. Drugs with these properties are expected to have the minimal side effects for cognitive functions of brain [4, 10, 12]. Prototypes of these noncompetitive NMDA blockers may be some minipeptides, which inhibit NMDARs preferentially when they are excessively open.

Synthetic сorticotropin-releasing factor-like peptide, mystixin-7 peptide (MTX), represents a novel class of (mystixins family) (molecular formula: C_51_H_84_N_12_O_9_S; MW: 1.041 kDa; and chemical structure: 4-anisoyl-arginyl-lysyl-leucyl-leucyl-thienyl-isoleucyl-leucinamide [14]). According to its amino acid structure, it relates to minipeptides [15].* In vivo* studies have shown that MTX has anti-inflammatory effects on nonnervous cells [16–19].

Our previous work revealed the MTX-induced marked neurotropic effects in brain slices. Peptide reversibly inhibited both the alpha-amino-3-hydroxy-5-methyl-4-isoxazolepropionic receptors (AMPARs) and NMDARs-mediated postsynaptic processes in a dose-dependent manner. These effects of MTX were reversible and activities of ionotropic glutamate receptors were restored after washing within 15–25 min. According to these findings, MTX peptide was characterized as noncompetitive and reversible inhibitor to both the AMPARs and NMDARs [20].

Likely, due to these properties, the peptide safely blocked ictal- and interictal-like events in dose- and time-dependent manner in the pentylenetetrazole (PTZ) model of ictogenesis in brain slices [21] and protected ionotropic glutamatergic mechanisms against oxygen-glucose deprivation [22]. Based on these data, we hypothesized that MTX has a wider range of protective action on a variety of disorders of the brain, which associated with the development of glutamate excitotoxicity. Therefore, the purpose of this study was to elucidate the protective effects of MTX in the model of glutamate excitotoxicity.

For this aim slice-based assay system was utilized to register the synaptic activity from rat olfactory cortex slices in combination with neurochemical treatment of MTX peptide.

Achievements of recent of decades in brain slice technology have made this experimental model exceptionally useful for examining pathophysiology of brain diseases in a tissue context. Brain slices maintain many aspects of* in vivo* physiology, including functional local synaptic circuitry with preserved brain architecture, while allowing good experimental access and precise control of the extracellular environment, making them ideal platforms for “dissection” of molecular pathways underlying neuronal dysfunction [23]. Importantly, these* ex vivo* systems permit direct treatment with pharmacological agents modulating these responses and thus provide surrogate therapeutic screening systems instead of the study on whole animals.

The olfactory cortex slices are optimal experimental objects. These slices are less traumatic because one of their surfaces (pial) remains intact. Moreover, the cutting surface is located on the inside of slices, keeping the normal function of cellular structures for the analysis of incoming sensory input. The morphological structures of slices are easily defined under slight magnifying. It allows localizing the stimulating and recording electrodes at selected points for the extracellular potentials recording. They are reliably identified as the separate exciting pre- and postsynaptic AMPA and NMDA components of synaptic response.

In present study we developed glutamate-induced excitotoxicity model on olfactory cortex slices, and with aid of this model we showed the ability of MTX peptide to protect the synaptic activity in the damaged brain slices. We revealed that peptide is an efficient and reliable protector against glutamate-induced excitotoxicity.

## 2. Materials and Methods

### 2.1. Animals

Wistar rats with body weight 100–150 g were obtained from vivarium (Pavlov Institute of Physiology, RAS, Saint Petersburg, Russia) and kept in animal room with controlled temperature (21 ± 1°C) and humidity (55%), with food and water ad libitum in a 12 h dark/light cycle. All efforts were made to minimize animal suffering and the number of animals used.

### 2.2. Preparation of Slices

Studies were performed on male Wistar rats with body weight 100–150 g (vivarium of the Pavlov Institute of Physiology, RAS) in compliance with ethical standards of the Directive 2010/63/EU of the European Parliament and of the Council of September 22, 2010, on the protection of animals used for scientific purposes. In this work methods of the slices preparation and their incubation were used as described in our previous publications [24–26]. Tangential slices of olfactory cortex 450–500 *μ*m thick were cut from the brain of male rats of Wistar line with body weight 100–150 g. The animals were decapitated by the guillotine. Using special surgical instruments brain was rapidly removed and placed on a metal table, cooled to +4°C, and covered with filter paper. With a scalpel along the midline, the brain was dissected into two halves and gently rolled over so that the olfactory cortex was upstairs. With the help a glass slide with support guide and special knife—“cutter” (Pavlov Institute of Physiology, RAN)—olfactory cortex slices of rat brain were prepared. These tools used to minimize injury to slices structures because our previous studies have shown that the use of vibroslicer leads to greater injury of the slices structures and worsens their vital activity [27].

The prepared slices were transferred to a glass vial with a brush and every slice preincubated for 1 h in 1 mL of artificial cerebrospinal fluid (aCSF) at 37°C, pH 7.21–7.24. The composition of aCSF was as follows (mM): NaCl, 124.0, KCl, 5.0, CaCl_2_, 2.6, KH_2_PO_4_, 1.24, MgSO_4_, 1.2, NaCHO_3_, 3.0, tris-HCL (pH 7.4), 23.0, and glucose, 10.0. aCSF was equilibrated with O_2_. The concentrations of Ca^2+^ and Mg^2+^ were optimized for maximal synaptic activity in olfactory cortex slices.

The duration of the entire procedure of slice preparation from the time decapitation and placing it in the incubation medium was 1 min. After placing the slice in a glass vial, gas atmosphere above a medium was replaced by oxygen for 1 min. The vials with slices were placed in a Warburg apparatus (Germany) with a frequency of 120 swings per min and a temperature of 37°C, where the slices were preincubated before being placed into the recording chamber. The incubation medium with slice was replaced by a fresh aCSF twice after 1 and 3 hours of preincubation in order to remove from medium the remnants of disrupted cells and their metabolites. The slices incubated at 37°C to restore full functioning of slice structures after their preparation. We early found that slicing using incubation medium warmed to near physiological temperature (37°C) greatly enhances slice quality without affecting intrinsic electrophysiological properties of the neuronal network [27]. The advantage of using such a method has been confirmed by recent studies of Huang and Uusisaari [28].

Osmolarity of aCSF was 295–305 mOsmo (OMT-5-01, “Burevestnic,” Russia). After preincubation, slice was transferred to the interface-recording chamber. Drug solutions were prepared in extracellular solutions and applied to slices by perfusion system at a constant rate (2 mL/min), controlled by the electronic device (Pavlov Institute of Physiology, RAS).

### 2.3. Electrical Stimulation and Recording Techniques

Extracellular field potentials (FPs) were evoked using platinum custom-made bipolar stimulating electrodes positioned onto the proximal part of the lateral olfactory tract (LOT), which is the main afferent input to the neurons of the olfactory cortex. Stimulation was applied as the rectangular pulses (duration: 0.1 msec, intensity: 1.2–1.5 V, and frequency: 0.003 Hz) using the stimulator ESU-1 (Russia).

The FPs were recorded using a glass microelectrode filled with 1 M NaCl with tip resistance 1–5 MΩ. Signals were registered with NTO-2 amplifier (Russia), digitized by analog-to-digital converter MD-32 (Russia), and stored on the computer. The recording point was located in the piriform cortex of the olfactory cortex slice. A silver reference electrode was located in the chamber floor.

**Figure 1:**
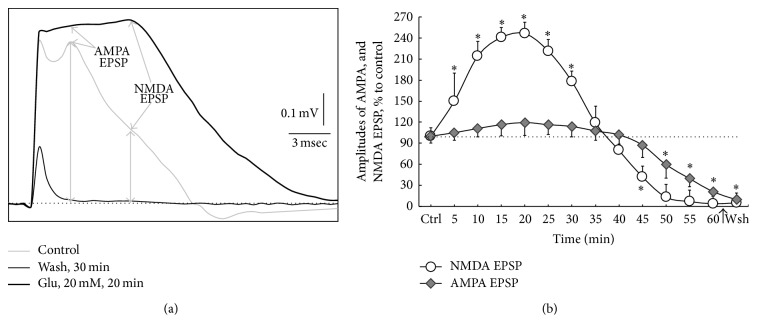
The model of glutamate excitotoxicity in the olfactory cortex slices. (a) Effects application of* L*-glutamate in toxic concentration (20 mM) on profiles FPs. Representative FPs were recorded in time points 1, 2, and 3, respectively, as indicated in (b). FPs are integral averaging potentials generated by neurons in slices processing by special computer program at four independent experiments performed in triplicate in different slices (*n* = 12* per* treatment condition). The dotted line indicates isoline. Arrows indicate AMPA and NMDA components of the EPSP. The vertical arrows from isoline to the peaks of the AMPA and NMDA EPSP show as conducted the measurements of their amplitudes. The captions about the conditions exposures to slices (legend beneath (a)) indicate time points of the registration FPs, corresponding to the points in (b), namely, (1) “Cntr,” (2) “Glu, 20 min,” and (3) “Wsh, 30 min.” Calibration as is indicated. At the FPs registration, the electronic device (Pavlov Institute of Physiology, RAS) for artefact-rejection was used. (b) Change of the AMPA and NMDA EPSP amplitudes at imitation glutamate excitotoxicity obtained at application of* L*-glutamate (20 mM) on the olfactory cortex slices. The *x*-axis—“Ctrl”: control values of AMPA and NMDA EPSP (without the glutamate), 15 min; thick arrow and “Wsh” indicate washout, 30 min. The dotted line indicates the control level corresponding to 100%. The duration application of* L*-glutamate on the slices for modeling of the glutamate excitotoxicity was 60 min. The results are expressed as percentage of control condition and represent means ± SEM of 4 independent experiments performed in triplicate in different slices and analyzed statistically by *U*-test, Wilcoxon-Mann-Whitney matched pairs signed-rank test. ^*∗*^ *p* ≤ 0.05, significantly different from control. *n* = 12, number of slices per time point performed in the repeated at least four independent experiments. At the FPs registration, the electronic device (Pavlov Institute of Physiology, RAS) for artefact-rejection was used. Note that* L*-glutamate induces the most significant hyperactivity of NMDARs with a maximum value at 20 min. Then the amplitudes of these components FPs progressively decreased and to 60th min were irreversibly blocked. The increase in AMPARs activity at action agonist was smaller, but with longer duration than NMDARs and after 40 min there was a decrease and irreversible blockade of these receptors' activity.

Typical FPs in the piriform cortex evoked by orthodromic stimulation of the LOT anterior part consist of two main components, namely, presynaptic (AP LOT) and postsynaptic: AMPA and NMDA EPSP. The components of FPs, their characteristics, pharmacological identification, and methods of measuring their amplitudes were described in detail earlier [24–26]. In present study we recorded and analyzed the changes amplitudes of both AMPA and NMDA EPSP. We estimated the amplitudes of these FP postsynaptic components from the isoline to the peak level as shown in Figure 1(a). The amplitudes of AMPA EPSP that we assessed within the 2 msec window centered at the peak of the response. Peak NMDA EPSP was measured as the average potential observed in the 8 msec window [29].

### 2.4. Drugs

Chimreactive Company (Russia) supplied chemical compounds for the preparation of aCSF;* L*-glutamate was received from Sigma (USA). MTX was provided by the University of California, Berkeley (USA). MTX was dissolved in aCSF immediately before testing. The prepared solution was filtered and kept in the thermostat at 37°C until use. The effect of MTX on the pH aCSF was tested. This is necessary to exclude possible side effects of the peptide interaction with incubation medium and determine its protective effects only. The optimal range of pH for normal activity of brain slices is 7.2–7.4. Addition of MTX to aCSF may evoke a modification of its pH and deteriorate the functioning of neurons in slice. By this reason pH of aCSF was measured (pH 150, Izmeritel, Russia) twice, namely, after preparation of MTX solution and after perfusion of slices by aCSF with MTX. In both conditions temperature of medium was maintained at 37°C. The pH of control aCSF (without MTX) was 7.24 ± 0.02 (*n* = 16* per* condition); after adding MTX it was 7.27 ± 0.02 (*n* = 7* per* treatment condition) and after perfusion of slices by medium with MTX it was 7.31 ± 0.02 (*n* = 7* per* treatment condition). Data obtained indicate that MTX solution was slightly alkaline, but it remained within the optimal range of pH throughout experiment.

*L*-Glutamate and MTX were delivered to slices via bath perfusion. To achieve an enzymatic inactivation of the MTX, the aCSF containing the peptide at a concentration of 250 mg/mL was treated with immobilized trypsin in a Sepharose 4B column loaded with activated CN-Br Sepharose 4 using the appropriate method (Pavlov Institute of Physiology, Russia). The solution containing MTX passed through the column of 25 mL volume by portions of 20 mL at 37°C.

### 2.5. The Design of the Experiment

We studied the protective effect of the MTX at concentrations 50 and 100 mg/mL. These concentrations of MTX were selected based on the previous studies, in which the minipeptide induced the most distinct neurotropic effects [20]. The protein was* ex tempore* dissolved in the incubation medium. At first, the slices perfused by control aCSF and FPs were recorded during 15 min. Among the components of the FPs we analyzed the amplitudes of postsynaptic components, AMPA and NMDA EPSP. These values were control for subsequent actions of MTX and* L*-glutamate actions on slices. Glutamate excitotoxicity was caused by perfusion of slices with medium containing 20 mM* L*-glutamate during 60 min in order to mimic glutamate excitotoxicity in the slices. Then slices were washed for 30 min.

In a series of experiments to study the protective effect of MTX (*n* = 16), FPs were recorded under control conditions and then during application of MTX (20 min) in slices. The LOT was stimulated under control conditions (without any drug) and during treatment with MTX with frequency 0.003 Hz.

### 2.6. Statistical Analyses

The statistical analyses of the changes in amplitudes of separate FP components were performed using the nonparametrical *U*-test, Wilcoxon-Mann-Whitney matched pairs signed-rank test (*p* ≤ 0.05). All the experiments were repeated four times containing three replicates per condition and time point. The quantified data in Figures 1, 3, and 4, are presented as percentage of control and are expressed as means ± SEM. In the figure captions *n* indicates the number of slices per treatment condition.

## 3. Results

### 3.1. Determination of Toxic Concentration of* L*-Glutamate

The glutamate-induced neurotoxicity is commonly used as experimental model of neuronal injury despite the fact that the NMDA receptors that cluster in dendrites spines [30] are likely to be critical in mediating more relevant pathophysiological processes [31]. However, there is no standard concentration of glutamate: its concentrations vary from several hundred to tens millimoles. Under normal conditions, extracellular glutamate concentration in the brain is 30 *μ*M [32, 33]. Glutamate concentration in astrocytes and neurons is higher by 1000 times [34]. The estimated concentration of glutamate at brain ischemia can attain or surpass 600 *μ*M [35]. After traumatic brain injury glutamate concentration in the extracellular space increased in the range of 50–100% compared to that before injury [36]. To induce totally the glutamate toxicity in hippocampal slices concentrations of exogenous agonist were used in the range 300 *μ*M–10 mM [37, 38].

Considering the above data to produce total hyperactivation of NMDARs and to impair the mechanism of glutamate reuptake in slices we had used exogenous glutamate in concentration of 20 mM.

### 3.2. The Model of Glutamate Excitotoxicity in the Olfactory Cortex Slices

We followed induced glutamate excitotoxicity in the slices in order to mimic the development of the acute stage of neurological diseases associated with glutamate toxicity and test the protective properties of MTX. For this purpose, slices were exposed to* L*-glutamate in toxic concentration of 20 mM for 60 min and then washed aCSF. The changes of both of AMPA and NMDA EPSP amplitudes were recorded and analyzed at stimulation of LOT. These data were needed in order to determine the activity of AMPARs and NMDARs-dependent mechanisms during the development glutamate-induced excitotoxicity.

Glutamate induced the increase in the amplitudes of both AMPA and NMDA EPSP. They peaked to 20 min of* L*-glutamate action as shown in Figure 1(a). Then the amplitudes of these FP components decreased and to 60 min were completely inhibited. Washing from the action of* L*-glutamate resulted in the blockade of AMPARs and NMDARs activity (Figure 1(a)). Minimal amplitude of the FP presynaptic component at the end of washing persisted (trace FP, marked in thin black line in Figure 1(a)).

Analysis of the temporal characteristics of the NMDA-mediated excitatory postsynaptic potentials revealed that the latency of these FP components increased to 1 ± 0.5 min after treatment of slices by* L*-glutamate. As for latency peak of AMPA EPSP accurately determining this parameter was technically impossible, which reflects the synaptic delay of the generation AMPA-dependent processes (Figure 1(a), trace FP, marked in black bold line).

Analysis of dynamics of change in the amplitudes AMPA and NMDA EPSP revealed that* L*-glutamate produced biphasic modifications of both AMPARs and NMDARs activities, which corresponded to a parabolic curve (Figure 1(b)). However, the amplitude/temporary characteristics of these curves were different for AMPARs and NMDARs activity. Therefore, the maximum increase activity was for NMDARs in average by 240% as compared with the control value. This increase lasted for an average of 20 ± 3 min (*n* = 12* per* time point); then the curve goes into descending phase; thereafter the amplitude of NMDA EPSP progressively decreased with duration 17 ± 2 min (intersection point with abscissa). Further, suppression of the activity NMDARs continued, reaching minimum values at 60 min. Blockade of these receptors was irreversible because at washing their activity was not restored (Figure 1(b)).

Unlike the NMDARs, the increase in AMPARs activity at action agonist was smaller, but with longer duration (40 ± 5 min, *n* = 12* per* point), than NMDARs (37 ± 2 min, *n* = 12* per* point). After 40 min, there was a decrease and irreversible blockade of the activity of these receptors (Figure 1(a)).

Thus, the acute phase of the development of glutamate-induced excitotoxicity was nonlinear for changing both of the AMPARs and NMDARs activity. These findings indicate that glutamate in toxic concentration affected both subtypes of ionotropic glutamate receptors, but the strongest action it had was on the NMDARs. The data provide a new way of looking at mechanisms leading to glutamate-induced excitotoxicity in nervous tissue and therefore may be important for the development of treatment strategies in protection of neurons in neurodegenerative diseases.

### 3.3. Effects of Different Concentrations of MTX on Modifications of the FPs Profiles Modification

In a previous study, it was shown that MTX modified the activities of AMPARs and NMDARs in a dose-dependent manner [20]. In this study, the duration registration of the protective effect was 20 min. This period was chosen to obtain the integral characteristics of the protective effect of MTX, depending on the concentration used.

**Figure 2:**
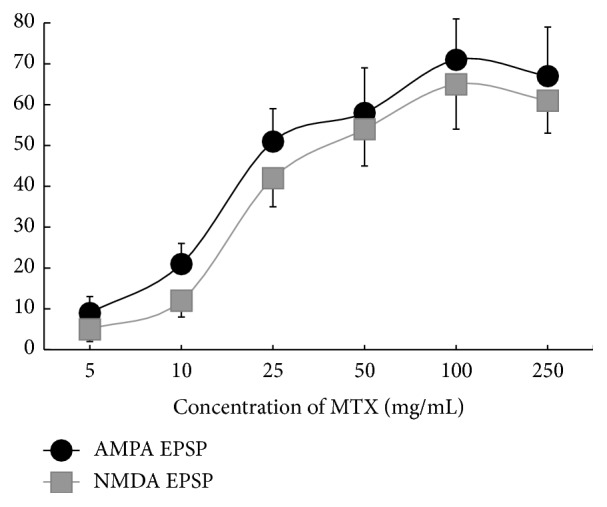
Protective effects of different concentrations MTX on the degree of preservation of activities of both AMPARs and NMDARs after exposure to glutamate in toxic concentration (20 mM). *y*-axis: amplitudes of AMPA and NMDA EPSP (mV) after pretreatment slices by MTX and further glutamate exposure (20 mM). *x*-axis: irregular. *n* = 7 for every point. Note that suppression of hyperactivation of AMPARs and NMDARs induced of* L*-glutamate was dependent upon the concentration of MTX at pretreatment of slices with this peptide.

With increasing concentrations of MTX in the bathing medium, the activities of both AMPARs and NMDARs mechanisms increased. Curves were sigmoid-shaped and congruent. The maximal protective effect was at concentration of 100 mg/mL for both receptor mechanisms (Figure 2). These results indicate that the protective effect of MTX may be enhanced for both AMPARs and NMDARs in a dose-dependent manner.

### 3.4. Neuroprotective Effect of MTX against Glutamate-Induced Excitotoxicity

This section presents data on the protective potential of MTX against glutamate excitotoxicity. Primarily we were interested in revealing the protective properties of MTX in the initial phase of the toxic action of* L*-glutamate at the time point 20 min. This time point has been crucial because it was the maximum activation of AMPARs and, especially, NMDARs (Figure 1(d)). The significance of this time interval in changing both AMPARs and NMDARs activities has been confirmed in other studies [25, 39].

In order to test this hypothesis* L*-glutamate in toxic concentration of 20 mM was applied on slices pretreated by MTX in concentration 50 mg/mL. Duration of* L*-glutamate action was 20 min. This time parameter has been used by us because according to the data presented in Figure 1(b) the maximum activating effects of agonist on both NMDARs and AMPARs were shown.

**Figure 3:**
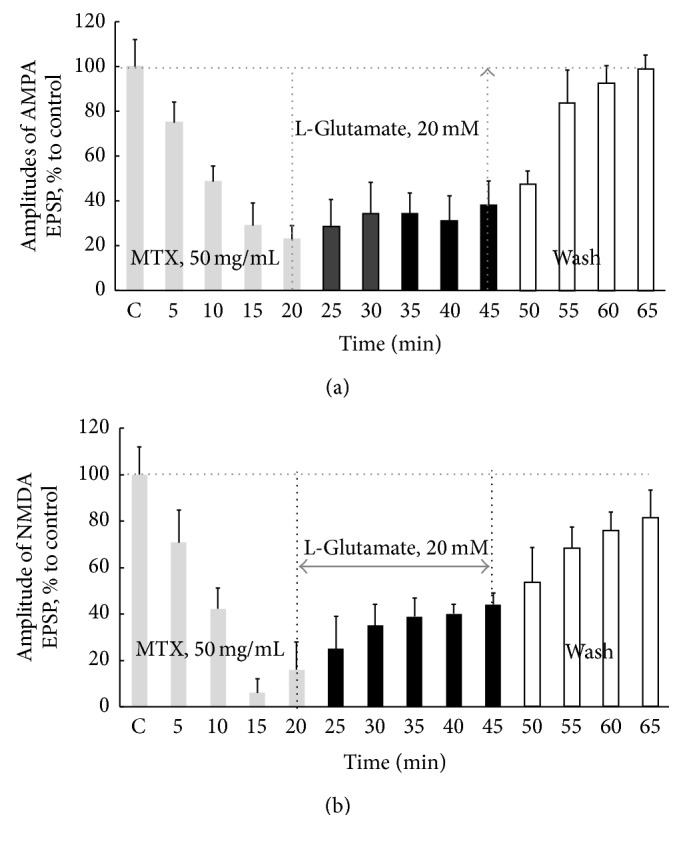
Neuroprotective effects pretreatment of slices by MTX at concentration 50 mg/mL on AMPARs (a) and NMDARs (b) activities at the action of* L*-glutamate in toxic concentration (20 mM). The left parts of the schedule (in (a) and (b)) show changes in the averaged amplitudes of AMPA and NMDA EPSP under the action MTX in concentration 50 mg/mL. Black columns indicate the beginning and the termination of* L*-glutamate action. Duration of* L*-glutamate action was 20 min. This time range has been used by us because according to the data presented in Figure 1(b) the maximum activating effects of glutamate in a toxic concentration of 20 mM on both NMDA and AMPA EPSP were at this duration of agonist exposure. “Wash, 20 min”: washing slices by aCSF during 20 min. Horizontal dotted line (in (a) and (b)) means the control values of the NMDA and AMPA EPSP amplitudes (without MTX and glutamate) before MTX application, and on the *x*-axis it is marked for “C, control” and the subsequent action of* L*-glutamate as well as at washing process. The data are presented as percentage of control condition and represent means ± SEM of five independent experiments performed in different slices and analyzed statistically by *U*-test, Wilcoxon-Mann-Whitney matched pairs signed-rank test. *n* = 16, number of slices per time point. ^*∗*^ *p* ≤ 0.05, significantly different from control. Note that suppression of hyperactivation of AMPARs and NMDARs induced* L*-glutamate at pretreatment of slices with this peptide. Recovery of the AMPARs and NMDARs activities at washout reached the control level.

Pretreatment of slices MTX induced modification activities of both AMPARs and NMDARs. These effects were presented in Figure 3.

At concentration of MTX 50 mg/mL the activity of AMPARs decreased by an average of 23% (Figure 3(a)).

Application of glutamate in toxic concentration on pretreated slices by MTX at dose of 50 mg/mL blocked the AMPARs hyperactivity (Figure 3(a)). However, at washing, their activity recovered as compared to control value (control before pretreatment—columns “MTX 50 mg/mL,” 100%, versus columns “Wash,” average, 99%).

A concentration of 50 mg/mL peptide in the bathing medium promoted improving its protective efficacy against glutamate excitotoxicity for NMDARs (Figure 3(b)). So, the average activity value of NMDARs in slices pretreated with MTX at a concentration of 50 mg/mL and followed by the action of glutamate was 39.3% (*U* = 11, *p* ≤ 0.05, and *n* = 16* per* treatment condition).

Noteworthy is the fact that after washing NMDARs activities progressively restored and through 20 min their activity was not different from the control level (columns “MTX 50 mg/mL” as compared with columns “wash 65 min”—75.5%) (Figure 3(b)).

### 3.5. An Enzymatic Treatment of MTX Peptide by Trypsin

In order to prove the specificity of the effects of MTX peptide it was enzymatically inactivated by trypsin and its activity was tested on slices immediately after this processing. We considered that after this treatment MTX should lose its neuroprotective properties if our hypothesis is correct. Trypsin cleaves peptides, containing amino acids, arginine and lysine, which are components of MTX.

**Figure 4:**
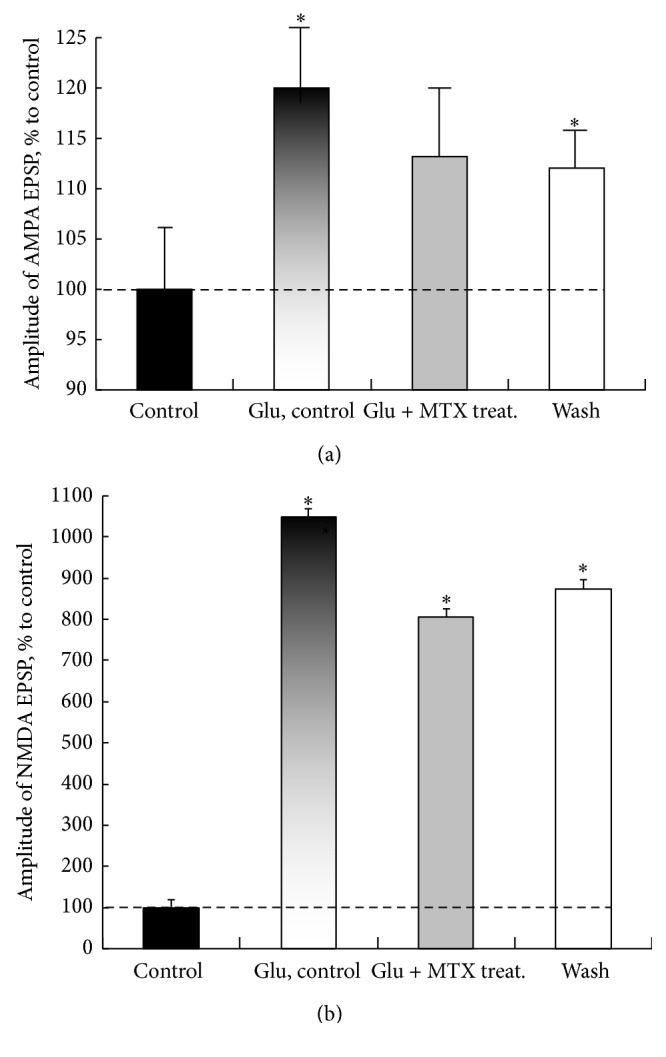
An enzymatic treatment of MTX (100 mg/mL) by trypsin resulted in a reduction of the peptide-mediated neuroprotection of AMPARs and NMDARs in slices of rat olfactory cortex. The dashed lines in (a) and (b) show control level of the AMPA and NMDA EPSP amplitudes. On the abscissa: control: the averaged values of the AMPA and NMDA EPSP amplitudes performed during 15 min; “Glu, ctrl”: effect of* L*-glutamate in toxic concentration 20 mM during 20 min; “Glu + MTX treat.”: effects of* L*-glutamate in toxic concentration 20 mM and subsequent action of the MTX pretreated during 25 min; and “wash”: washout, 20 min. The data are expressed as percentage of control value and represent means ± SEM analyzed statistically by *U*-test, Wilcoxon-Mann-Whitney matched pairs signed-rank test. ^*∗*^ *p* ≤ 0.05, significantly different from control. *n* = 12, number of slices per column. Statistically significant differences as compared to control are indicated by asterisks; *U*-test, *p* ≤ 0.05. *n* = 12 for every point. Note that the enzymatic pretreatment of MTX resulted in a decrease (statistically insignificant) in the amplitudes of AMPA EPSP but does not completely block theirs. The same values of responses persisted after washout. Hyperactivity of NMDARs to glutamate action and subsequent treatment of slices by MTX significantly decreased but remained increased compared with the control level.

The proteolytic cleavage of MTX (100 mg/mL) resulted in an insignificant (not statistically significant) decrease of the AMPA EPSP amplitude compared with the action of* L*-glutamate in the toxic concentration of 20 mM (column “Glu, ctrl”: 120.5% versus column “Glu + MTX treat.” denatured: 113.7%, *U* = 21, *p* ≥ 0.05, and *n* = 7* per* treatment condition). When removing the enzymatically treated MTX from bathing medium AMPA EPSP amplitude remained the same as under the action of* L*-glutamate (column “Glu, ctrl”: 120.5% versus column “wash”: 112.4%, *U* = 24, *p* ≥ 0.05, and *n* = 7* per* treatment condition) (Figure 4(a)).

Pretreatment of slices of enzymatically treated MTX (100 mg/mL) and subsequent action of* L*-glutamate expressed statistically significant decrease in the amplitude of NMDA EPSP (column “Glu, ctrl”: 1050.8% versus column “Glu + MTX treat.” denatured: 806.9%, *U* = 24, *p* ≤ 0.05, and *n* = 7* per* treatment condition) (Figure 4(b)). At washing similar amplitudes ratio for NMDA EPSP persisted (column “Glu, ctrl”: 1050.8% versus column “wash”: 875.5%, *U* = 29, *p* ≤ 0.05, and *n* = 7* per* treatment condition) (Figure 4(b)).

These findings indicate that the enzymatic pretreatment of MTX reduced but did not completely eliminate glutamate-induced hyperactivation AMPARs. Unlike the AMPARs, denatured MTX reduced overactivation the NMDARs. It is possible that these effects are produced by the remaining fragments of MTX leucyl-leucyl-thienyl-isoleucyl-leucinamide.

Together, these data indicate that MTX peptide has protective properties against glutamate excitotoxicity in slices and support the hypothesis concerning the specificity of MTX protective effects.

## 4. Discussions

The present study demonstrated that glutamate-induced synaptic impairment was fully prevented by MTX peptide, as a noncompetitive AMPARs and NMDARs antagonist. We found that the protective efficacy of MTX in this model neuropathology was enhanced with increasing its concentration in the bathing medium. In our opinion, this dependence has the following explanation. The glutamate-induced toxicity triggered by activation of somatic receptors requires a high concentration of agonist. Therefore, the protection using competitive antagonists like D-APV or CGS-19755 can only be achieved by very high concentrations of these drugs [40].

Moreover, differences in protective effects of MTX can be explained by different localization of NMDARs and AMPARs clusters on neurons in olfactory slices. Probably molecules of MTX at first acted on AMPARs located on the dendrites and then impacted on NMDARs located on the soma of neurons. Likely, features of localization of these receptors are critical in mediating the different pathophysiological processes [31].

The protective effect of MTX was maintained and upon removal of peptide from bathing medium that indicated a sustained recovery of AMPA- and NMDA-dependent mechanisms. Earlier, it has been found that MTX interaction with the NMDARs was longer and stronger [20]. The difference in the degree of MTX interaction with AMPARs and NMDARs was probably explained by the different composition of the subunits of these receptor complexes. Future experiments will be designed to test this assumption.

Currently there are many agents, which prevent or modulate glutamate receptor hyperactivation in order to control cell destruction. Therefore, MK-801, AP5, and other noncompetitive and competitive antagonists of NMDARs prevented glutamate-induced synaptic damage [41]. These data show that NMDARs are implicated in glutamate-induced injury in slices and are the key mechanisms of glutamate-induced excitotoxicity.

However, the question on the involvement of AMPARs in glutamate-induced toxicity remains open. In order to elucidate this question, we analyzed the change in the activity of AMPARs exposed to* L*-glutamate in toxic concentrations to mimic acute glutamate excitotoxicity. We revealed that* L*-glutamate causes a slight increase in activity of AMPARs. During the prolonged action of* L*-glutamate the AMPARs activity as well as NMDARs activity decreased and then was blocked irreversibly. These findings indicate that sensitivity of the AMPARs and NMDARs to toxic effect of glutamate is different. Moreover, AMPARs sensitivity to the action of the agonist is less pronounced than the NMDARs. Additionally, the different sensitivity of ionotropic glutamate receptors to glutamate was explained by the different composition of the subunits forming these receptor complexes [42, 43].

We analyzed the MTX protective effect on these receptors because the MTX as noncompetitive antagonist inhibited the AMPARs activity [20]. Indeed, MTX protected AMPARs as well as NMDARs activity against glutamate-induced toxic disturbance. These findings indicate that the protective potential of MTX increased compared with “classical” noncompetitive and competitive antagonists of NMDARs. This conclusion is supported by data on the protection of ionotropic glutamate receptors by using MTX in pentylenetetrazole model of ictogenesis. In this model of the glutamate excitotoxicity, the peptide reliably blocked both ictal and interictal epileptic-like activity in slices [21] and in another model has protected nerve cells against oxygen-glucose deprivation [22]. Together these data indicate that MTX possesses a broader protective potential. It should be noted that neuroprotective potential of MTX is enhanced by anti-inflammatory effects that has been proven in special studies [16–19, 44].

Molecular mechanisms of protective properties of MTX are not yet clear. We hypothesize that glutamate can induce irreversible AMPARs and NMDARs disturbance in slices of olfactory cortex via two mechanisms. One of them is connected with the involving of the binding of glutamate with NMDARs that lead to receptor-mediated excitotoxicity and that interaction can be blocked by MTX. The other mechanism is promoted by glutamate-induced reversal of its uptake, although in our studies glutamate transport was not evaluated. Besides, we cannot exclude the possibility that* L*-glutamate may also act via metabotropic glutamate receptors. Future experiments will be designed to test these hypotheses about the involvement of MTX in these mechanisms.

To understand the mechanisms of MTX action, it should be noted that this peptide has the structural similarity to a fragment derived from the COOH-terminal G-domain of the laminin-*α*1 chain [17]. It is known that laminins are heterotrimeric basement membrane glycoproteins and are essential molecular constituents of all basement membranes of multicellular organisms. In the CNS, laminins are involved in the formation and maintenance of the blood brain barrier and in trauma process [45, 46]. Neurotropic effects of laminins are unknown. However, derived laminins have obvious neurotropic properties. For example, the KDI peptide (Lys-Asp-Ile) derived from gamma 1-laminin tripeptide induced a reduction of glutamate excitotoxicity by inhibiting AMPARs and NMDARs in a dose-dependent and noncompetitive manner [45, 47]. It should be noted that MTX acted on AMPARs analogously as on NMDARs in our model of glutamate-induced excitotoxicity. Hence, these data indicate not only structural, but also functional similarities between MTX peptide and laminins.

The specificity of the effects of MTX was demonstrated by its enzymatic treatment by trypsin. Trypsin cleaves peptides containing amino acids, arginine and lysine. Such processing was accompanied by a reduction of protective potential of MTX peptide. These experimental conditions imitated the MTX degradation under the action of extracellular peptidases and confirmed the idea about specificity of protective effects caused by MTX in model of the glutamate-induced toxicity. Moreover, it is obvious that for constructing derivatives of this minipeptide limiting the number of amino acids required to maintain its protective potential should be considered.

Hyperactivation of glutamate receptors, loss of energy supply leading to transmembrane ion gradients disruption, and altered transport function are common features of many neurological disorders, which involve glutamate excitotoxicity, such as stroke, anoxia, amyotrophic lateral sclerosis, and epilepsy [8]. Hence, understanding the mechanisms involved in glutamate-induced toxicity, as well as searching compounds that can prevent its effects, may have therapeutic significance in treatment of neurological diseases involving glutamate excitotoxicity.

## 5. Conclusions

In summary, MTX peptide reliably protects the functioning of both NMDARs and AMPARs against glutamate-induced damage. The data presented lead us to hypothesize that MTX is a multifaceted protector for the specialized synapses in the stressed central nervous system. We hope that our results concerning the protective effects of MTX peptide will contribute to the elaboration of effective therapeutic medications for the treatment of neurological diseases mediated by glutamate excitotoxicity development.

## Abbreviations

MTX: Mystixin-7
aCSF: Artificial cerebrospinal fluid
LOT: Lateral olfactory tract
FP: Field potential
EPSP: Excitatory postsynaptic potential
NMDA: * N*-Methyl-D-aspartate
AMPA: Alpha-amino-3-hydroxy-5-methyl-4-isoxazolepropionic.
